# Modulation of medial prefrontal cortical activity using in vivo recordings and optogenetics

**DOI:** 10.1186/1756-6606-5-36

**Published:** 2012-10-08

**Authors:** Guangchen Ji, Volker Neugebauer

**Affiliations:** 1 Department of Neuroscience & Cell Biology, The University of Texas Medical Branch, 301 University Blvd, Galveston, TX, 77555-1069, USA

**Keywords:** Optogenetics, Medial prefrontal cortex, Infralimbic, Prelimbic, Pyramidal cells, Single-unit recording, Electrophysiology, Cognitive, Emotion

## Abstract

**Background:**

The medial prefrontal cortex (mPFC) serves major executive functions. mPFC output to subcortical brain areas such as the amygdala controls emotional processing and plays an important role in fear extinction. Impaired mPFC function correlates with extinction deficits in anxiety disorders such as PTSD and with cognitive decision-making deficits in neuropsychiatric disorders and persistent pain. Controlling mPFC output is a desirable therapeutic goal in neuropsychiatric disorders but functional differences of cell types (pyramidal cells and interneurons) and regions (infralimbic and prelimbic) represent a challenge. This electrophysiological study used optogenetics for the cell- and region-specific modulation of mPFC pyramidal output in the intact anesthetized animal.

**Results:**

Extracellular single-unit recordings were made from infralimbic (IL) pyramidal cells, IL interneurons and prelimbic (PL) pyramidal cells 2–3 weeks after intra-IL injection of a viral vector encoding channel rhodopsin 2 (ChR2) under the control of the CaMKII promoter (rAAV5/CaMKIIa-ChR2(H134R)-EYFP) or a control vector that lacked the ChR2 sequence (rAAV5/CaMKIIa-EYFP). Optical stimulation with laser-generated blue light pulses delivered through an optical fiber to the IL increased spontaneous and evoked action potential firing of ChR2 expressing IL pyramidal cells but had no effect on IL interneurons that were distinguished from pyramidal cells based on their higher firing rate and shorter spike duration. Optical activation of IL pyramidal cells also inhibited PL pyramidal cells, suggesting that IL output controls PL output. The effects were light intensity-dependent and reversible. Confocal microscopy confirmed ChR2-EYFP or control vector expression in mPFC pyramidal cells but not in GABAergic cells.

**Conclusions:**

The novelty of our study is the analysis of optogenetic effects on background and evoked activity of defined cell types in different mPFC regions. The electrophysiological in vivo results directly demonstrate the optogenetic modulation of mPFC activity in a region- and cell type-specific manner, which is significant in conditions of impaired mPFC output.

## Background

The medial prefrontal cortex (mPFC) serves executive functions that are essential for selecting appropriate and inhibiting inappropriate actions. Prefrontal cortex dysfunction has been identified as a key neurobiological correlate of cognitive inflexibility and behavioral disinhibition associated with neuropsychiatric disorders such as drug addiction, obsessive-compulsive disorder, anxiety disorders and schizophrenia [1-8]. The important role of the mPFC in top-down cognitive control mechanisms is particularly well documented in experimental models of behavioral “extinction” of negative emotions [9-12].

The infralimbic region of the mPFC inhibits amygdala output to suppress (“extinguish”) aversive behaviors [10,13-18]. Increased thickness and activity of the mPFC correlate with successful extinction of negative emotions [19-22] whereas decreased activity has been implicated in cognitive control deficits in models of extinction [23-26] and behavioral disinhibition [2]. The concept of behavioral extinction forms the neurobiological basis for certain cognitive behavioral therapies in emotional-affective disorders [11,27] and chronic pain [28]. Extinction deficits have been proposed as a mechanism of the persistence of pain and its negative affective dimension [29]. Abnormalities in the mPFC are found in human pain patients and in animal pain models [30,31]. Our studies showed that amygdala-driven abnormal inhibition and decreased output of mPFC pyramidal cells contribute to pain-related impaired decision-making [32]. The underlying mechanism is feedforward inhibition of mPFC pyramidal cell output [32-34].

Therefore, increasing mPFC output to engage cognitive control systems is a desirable goal in conditions and disorders that are associated with decreased mPFC activity, such as anxiety [35-37], depression [38,39] and pain [32]. Differential roles of infralimbic and prelimbic mPFC regions [23], diversity of excitatory and inhibitory neurons [40], and complex pharmacology and neurochemistry [41] present a challenge for the selective control of mPFC activity. Recently developed optogenetic tools allow the activation or inhibition of distinct neuronal cell types within defined brain regions [42,43] but few studies have analyzed the effects of optogenetic manipulation of mPFC function. Optogenetic activation, but not inhibition, of excitatory mPFC cells inhibited unconditioned social exploratory behavior and fear conditioning [44] and had antidepressant-like effects [38]. Activation of GABAergic mPFC interneurons impaired operant delayed alternation performance [45] but rescued impaired social behavior due to abnormal mPFC excitation [44]. The neuronal effects of optical activation were confirmed using patch-clamp recordings in brain slices [44,45] and activity markers [38]. Electrophysiological effects of optogenetic manipulations in the mPFC on neuronal activity in vivo were addressed only recently in studies that recorded multiunit activity [44,46].

The novelty of the present study is the use of single-unit recordings in the intact animal to determine the effect of optical activation of infralimbic pyramidal output on spontaneous and evoked responses of infralimbic pyramidal cells, infralimbic interneurons and prelimbic pyramidal cells. This work is significant because infralimbic output to subcortical brain areas such as the amygdala plays a key role in fear extinction that might be utilized in the treatment of anxiety disorders such as PTSD [17,23,47].

## Methods

### Animals

Adult male Sprague Dawley rats (250–350 g) were housed in standard plastic cages (40 × 20 cm) in a temperature-controlled room and maintained on a 12-h day/night cycle. Water and food were available without restriction. All experimental procedures were approved by the Institutional Animal Care and Use Committee at The University of Texas Medical Branch and conform to the guidelines of the International Association for the Study of Pain and of the National Institutes of Health.

### Optogenetics

rAAV5/CaMKIIa-ChR2(H134R)-EYFP and a control vector that lacked the ChR2 sequence (rAAV5/CaMKIIa-EYFP) from the Karl Deisseroth laboratory [48-50] were produced by the Vector Core Facility at The University of North Carolina, Chapel Hill. Rats were anesthetized with 2–4% isoflurane, and a small unilateral craniotomy was performed. Using a 10 μl Hamilton syringe, 1 μl virus suspension was delivered at a rate of 0.1 μl/min into the infralimbic cortex (stereotaxic coordinates: 3.2 mm anterior to bregma; 0.8 mm lateral to midline; depth, 5.0-5.5).

### Electrophysiology

#### Anesthesia and surgery

2–3 weeks after virus injection the animal was prepared for electrophysiological recordings as described in detail in our previous studies [32,51]. The animal was anesthetized with pentobarbital sodium throughout the experiment (induction, 50 mg/kg, ip; maintenance, 15 mg· kg^-1^·h^-1^, iv), paralyzed with pancuronium (0.3 mg/h, iv) and artificially ventilated (3–3.5 ml; 55–65 strokes/min). End-tidal CO2 levels (kept at 4.0%), heart rate, and ECG pattern were monitored continuously. Core body temperature was maintained at 37°C by means of a homeothermic blanket system. These measures ensured a constant internal state of body functions. The animal was mounted in a stereotaxic frame (David Kopf Instruments), and a small unilateral craniotomy was performed to allow the insertion of the recording electrode and optical fiber.

#### Single-unit recording

Extracellular recordings were made from single neurons in the prelimbic and infralimbic part of the mPFC with glass-insulated carbon filament electrodes (4–6 MΩ) using the following stereotaxic coordinates: 3.0-3.2 mm anterior to bregma; 0.5–1.0 mm lateral to midline; depth, 3.0–4.6 mm (prelimbic) and 4.6-5.6 mm (infralimbic). Recorded signals were amplified, band-pass filtered (300 Hz – 3 kHz), displayed on analog and digital storage oscilloscopes, fed into a window discriminator (World Precision Instruments), digitized (1401 plus interface, Cambridge Electronic Design), and recorded on a Pentium 4 personal computer (PC) using Spike2 software (version 4; Cambridge Electronic Design) to analyze single-unit activity.

#### Identification of mPFC neurons

An individual neuron was identified by the configuration, shape, and height of the recorded action potentials (spikes) that occurred spontaneously (background activity) or in response to mechanical (tissue compression) search stimuli (evoked responses). mPFC pyramidal neurons can be distinguished from interneurons based on their broader action potential waveform (peak-to-valley >500 μs) and lower baseline discharge rate (<10 Hz) as described before [32,51]. Spikes were detected and recorded based on the waveform signal that crossed a trigger level and matched a pre-set shape or template, which was created for the individual neuron at the beginning of the recording period using Spike2 software. Only those neurons were included in this study whose spike configuration remained constant (matching the template) and could be clearly discriminated from activity in the background throughout the experiment, indicating that the activity of one and the same one neuron was measured.

#### Experimental protocol

##### Background and evoked activity

Spontaneous activity (in the absence of intentional stimulation) and responses evoked by brief (10 s) mechanical test stimuli of innocuous (300 g/30 mm^2^) and noxious (2000 g/30 mm^2^) intensities were recorded. Mechanical stimuli (compression) were applied to the knee joint by means of a calibrated forceps equipped with a force transducer, whose output signal was amplified, displayed in grams on a liquid-crystal display screen, digitized by the CED interface, and recorded on the Pentium PC for on- and offline analysis as described before [32,51]. For the analysis of net evoked activity, background activity in the 15-s time period preceding the 15-s stimulus was subtracted from the total activity during stimulation.

##### Optical stimulation

After a control period of 20–30 min the effect of optical stimulation of ChR2-expressing cells on background and evoked activity of an individual neuron was recorded. Laser-generated blue light pulses (15 ms; frequency, 10 Hz; intensities, 1, 5, 10 mW) were delivered to the infralimbic mPFC through an optical fiber (200 μm diameter) coupled to a 473-nm laser (ThorLabs). Stimulus parameters of the laser were controlled with a pulse generator (Grass Instruments). The fiber was attached to a stereotaxic cannula holder (Kopf Instruments). Several hours before the start of the electrophysiological recordings, the optical fiber was lowered vertically into the infralimbic mPFC at the site of the virus vector injection (see above). Duration of optical stimulation was 40–60 s to determine effects on background activity in the absence of peripheral mechanical stimuli, and 1–2 min while mechanical stimuli were applied to measure the effect on evoked activity.

##### Verification of recording and stimulation sites and virus expression

At the end of each experiment, the recording site in the mPFC was marked by injecting direct current (250 μA for 3 min) through the carbon-filament glass electrode. Brains were removed and fixed in 4% paraformaldehyde for 6–12 h; then they were placed in 30% sucrose for 48–72 h before they were frozen-sectioned at 50 μm. For verification of virus expression, brain slices were washed in PBS and mounted on gelatin-coated slides treated with fluorescent-mounting media. To verify absence of virus expression in GABAergic cells, brain slices were rinsed in PBS and placed in 10% normal goat serum (NGS) for 1 hour. Tissues were rinsed in PBS several times and incubated in primary antibody (mouse monoclonal antibody, anti-GAD67, 1G10.2, Millipore, dilution 1:500) for 48 hours at 4Â°C. Sections were washed again in PBS and transferred to TRITC conjugated goat anti-mouse IgG (AP503R, Millipore, dilution 1:100) for 1 h at 37Â°C. After three rinses in PBS, sections were mounted on slides treated with fluorescent-mounting media. Expression of ChR2-eYFP and GABA was then examined using a Zeiss laser-scanning confocal microscope. For verification of the position of the optical fiber tip and the recording/lesion site, sections were mounted on gel-coated slides, stained with hematoxylin and eosin, and cover slipped.

##### Data analysis and statistics

Single-unit action potentials were analyzed offline from peristimulus rate histograms using Spike2 software (version 4; Cambridge Electronic Design). Background and evoked activity was expressed as spikes/s (Hz). Background activity preceding each stimulus was subtracted from the total activity during stimulation to calculate net evoked activity. All averaged values are given as the mean ± SE. Statistical analysis was performed on the raw data (spikes/s). For multiple comparisons, one-way ANOVA was used with appropriate posttests. Statistical significance was accepted at the level *P <* 0.05. GraphPad Prism 3.0 software was used for all statistical analyses.

## Results

### Channel rhodopsin 2 (ChR2) expression in infralimbic mPFC pyramidal cells

**Figure 1:**
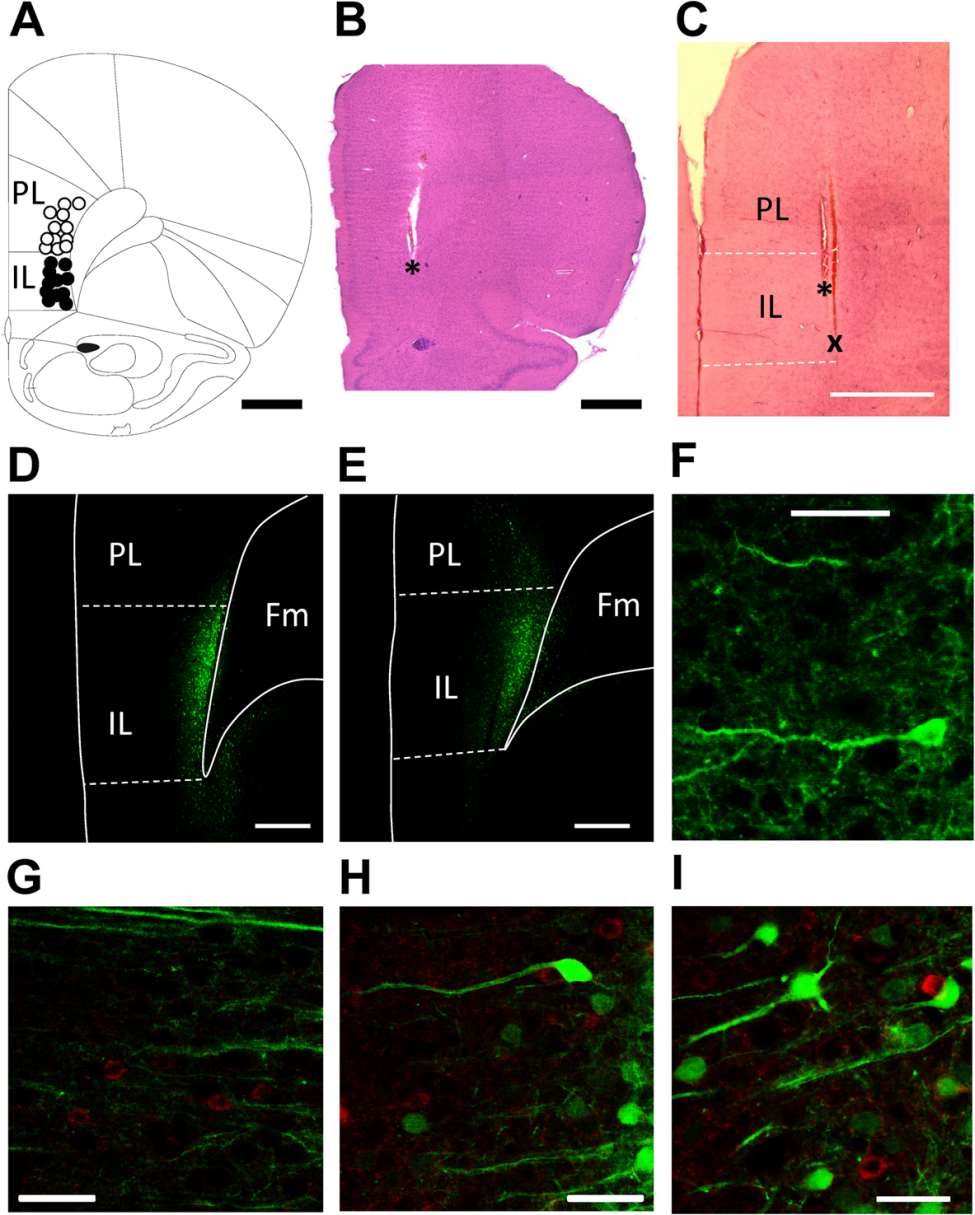
**Recording and stimulation sites and viral vector expression in the mPFC.** (**A**) Recording sites of pyramidal cells in the infralimbic (IL, n = 11) and prelimbic (PL, n = 12) cortex. (**B**) Position of the optic fiber in the IL (* indicates tip). (**C**) Positions of the optic fiber (*) and the recording site (x) in the IL. (**A**-**C**) Scale bars, 1 mm. (**D**) Yellow fluorescent protein (YFP) labeling indicates ChR2 expression restricted largely to IL (deep layers) following rAAV5/CaMKIIa-ChR2(H134R)-EYFP injection into IL. (**E**) Expression of control vector that lacked the ChR2 sequence (rAAV5/CaMKIIa-EYFP). (**D**, **E**) Scale bars, 500 μm. Fm, forceps minor. (**F**) Example of a ChR2 expressing pyramidal cell in IL. Scale bar, 50 μm. (**G**-**I**) Lack of ChR2/viral vector co-expression (green) in GAD positive (GABAergic) interneurons (red). Scale bars, 50 μm. All sections are taken at the level of 3.2 anterior to bregma.

Stereotaxic injection of a viral vector encoding channel rhodopsin 2 (ChR2) and yellow fluorescent protein (YFP) under the control of the CaMKII promoter (rAAV5/CaMKIIa-ChR2(H134R)-EYFP) led to the expression of ChR2 in infralimbic pyramidal cells (Figure 1D,F) but not in GABAergic interneurons (Figure 1G-I). The spread of rAAV5/CaMKIIa-ChR2(H134R)-EYFP (Figure 1D) and of a control vector lacking the ChR2 sequence (rAAV5/CaMKIIa-EYFP; Figure 1E) was similar and largely restricted to the deep layers of the infralimbic mPFC.

### Effect of optical stimulation on mPFC neurons

Extracellular single-unit recordings were made from 23 pyramidal cells (see recording sites in Figure 1A; 11 infralimbic, IL; 12 prelimbic, PL) and from 5 infralimbic interneurons in rats injected with rAAV5/CaMKIIa-ChR2(H134R)-EYFP. For optical stimulation the fiber was positioned in the IL (Figure 1B and C).

### Background activity

**Figure 2:**
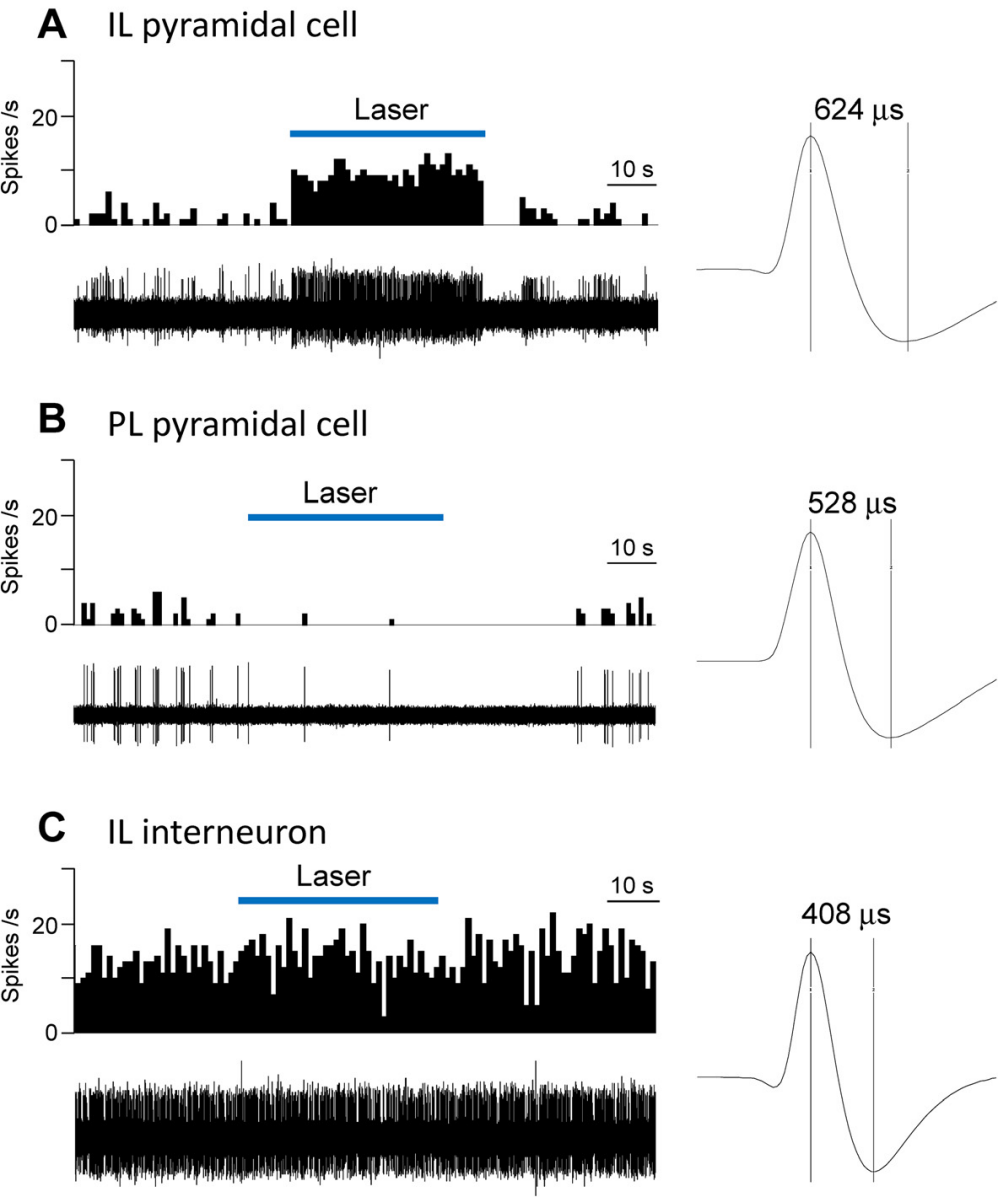
**Effects of optical activation on background activity of individual mPFC neurons**. Extracellular single-unit recordings in anesthetized rats. (**A**) Laser stimulation in IL activated an IL pyramidal cell. (**B**) Optical stimulation in IL inhibited a PL pyramidal cell. (**C**) Laser stimulation had no effect on an IL interneuron. Peristimulus time histograms (PSTHs, bin width, 1 s) show number of action potentials (spikes/s) before, during and after laser stimulation (5 mW, 10 Hz). Original recordings of action potentials are shown below PSTHs. Spike 2 software was used to isolate and count single-unit activity based on waveform signal that crossed a trigger level and matched a pre-set shape or template (see Methods). Traces on the right show individual spikes; numbers indicate peak-to-valley spike width.

**Figure 3:**
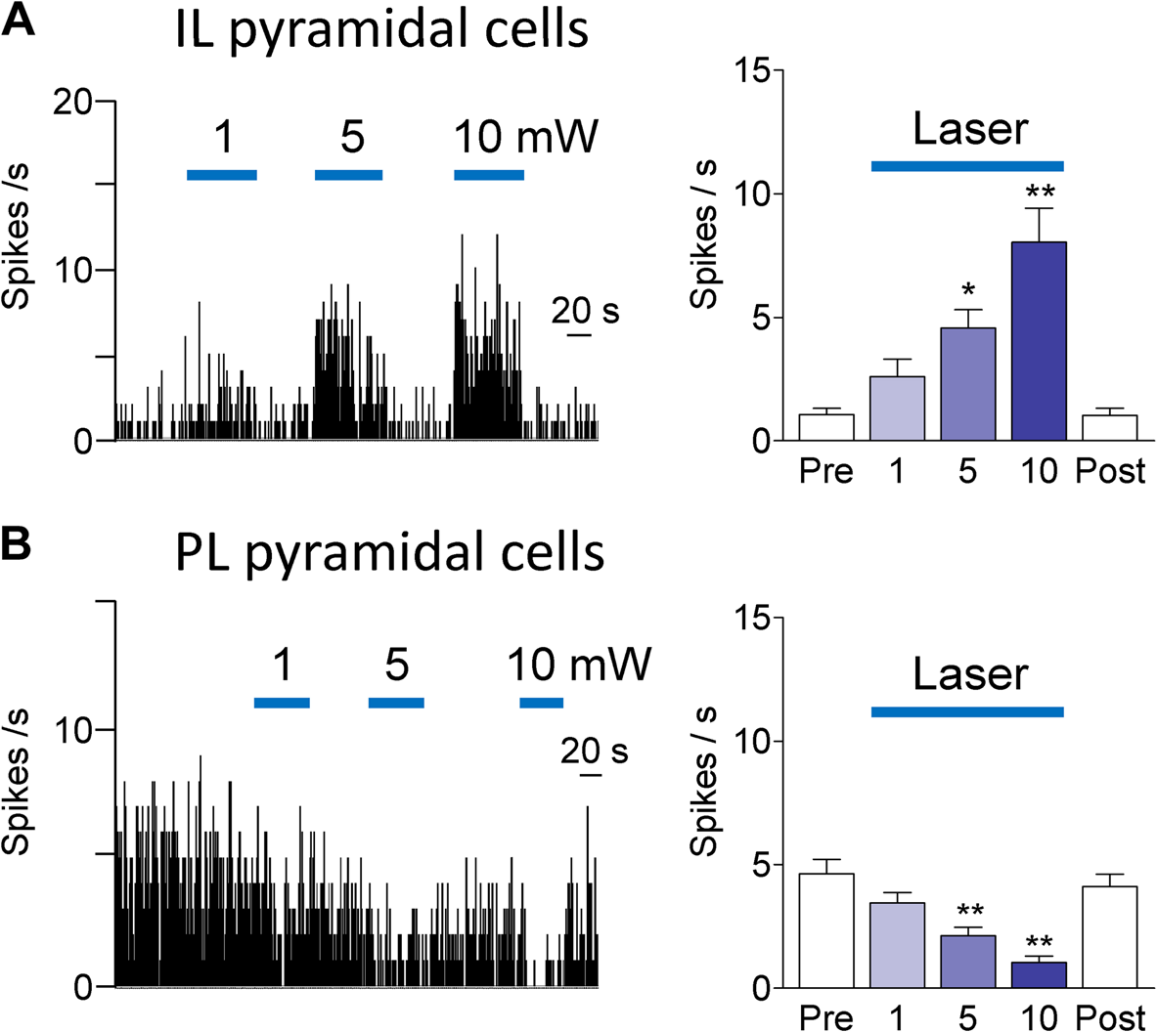
**Effects of optical activation on background activity.** Effect of optical stimulation with different light intensities (1, 5, 10 mW; 10 Hz) for 40–60 s on background activity of mPFC pyramidal cells. (**A**) Intensity-dependent activation of IL pyramidal cells by optical stimulation in IL. Left, Peristimulus time histograms (bin width, 1 s) show background activity of an individual neuron. Right, Bar histograms show summary data (means ± SE) for the sample of IL pyramidal cells (n = 9). Background activity was recorded before (Pre), during (1, 5 and 10 mW) and after (Post) optical stimulation. (**B**) Intensity-dependent inhibition of PL pyramidal cells by optical stimulation in IL. Left, PSTHs show background activity of an individual neuron. Right, Bar histograms show summary data (means ± SE) for the sample of PL pyramidal cells (n = 12). *, ** P < 0.05, 0.01 (compared to control before stimulation “Pre”, Dunnett’s multiple comparison tests).

The effect of optical stimulation (5 mW, 10 Hz, for 40–60 s) on background activity in the absence of peripheral mechanical stimuli (see Methods) in individual mPFC neurons is shown in Figure 2. Optical stimulation of ChR2 expressing neurons in IL increased activity in an IL pyramidal cell (Figure 2A) but inhibited background activity in a PL pyramidal cell (Figure 2B) and had no effect on an IL interneuron (Figure 2C). Compared to pyramidal cells, the interneuron showed higher levels of background activity and had a narrower spike width, which are distinguishing characteristics as described in our previous studies [32,51]. The differential effects of optical activation (laser pulses of 1–10 mW, 10 Hz, for 40–60 s) of ChR2 expressing neurons in IL on pyramidal cells in IL and PL are summarized in Figure 3. 9 of 11 IL pyramidal cells were excited (Figure 3A) whereas 2 IL pyramidal cells were inhibited (not shown). Background activity of all 12 PL pyramidal cells was inhibited (Figure 3B). The excitatory and inhibitory effects of optical stimulation were intensity-dependent and reversible. Laser intensities of 5 and 10 mW produced significant effects (P < 0.05 and 0.01, Dunnett’s multiple comparison tests, Figure 3). Fast-spiking interneurons (n = 5) in IL were not affected by optical stimulation.

### Evoked activity

**Figure 4:**
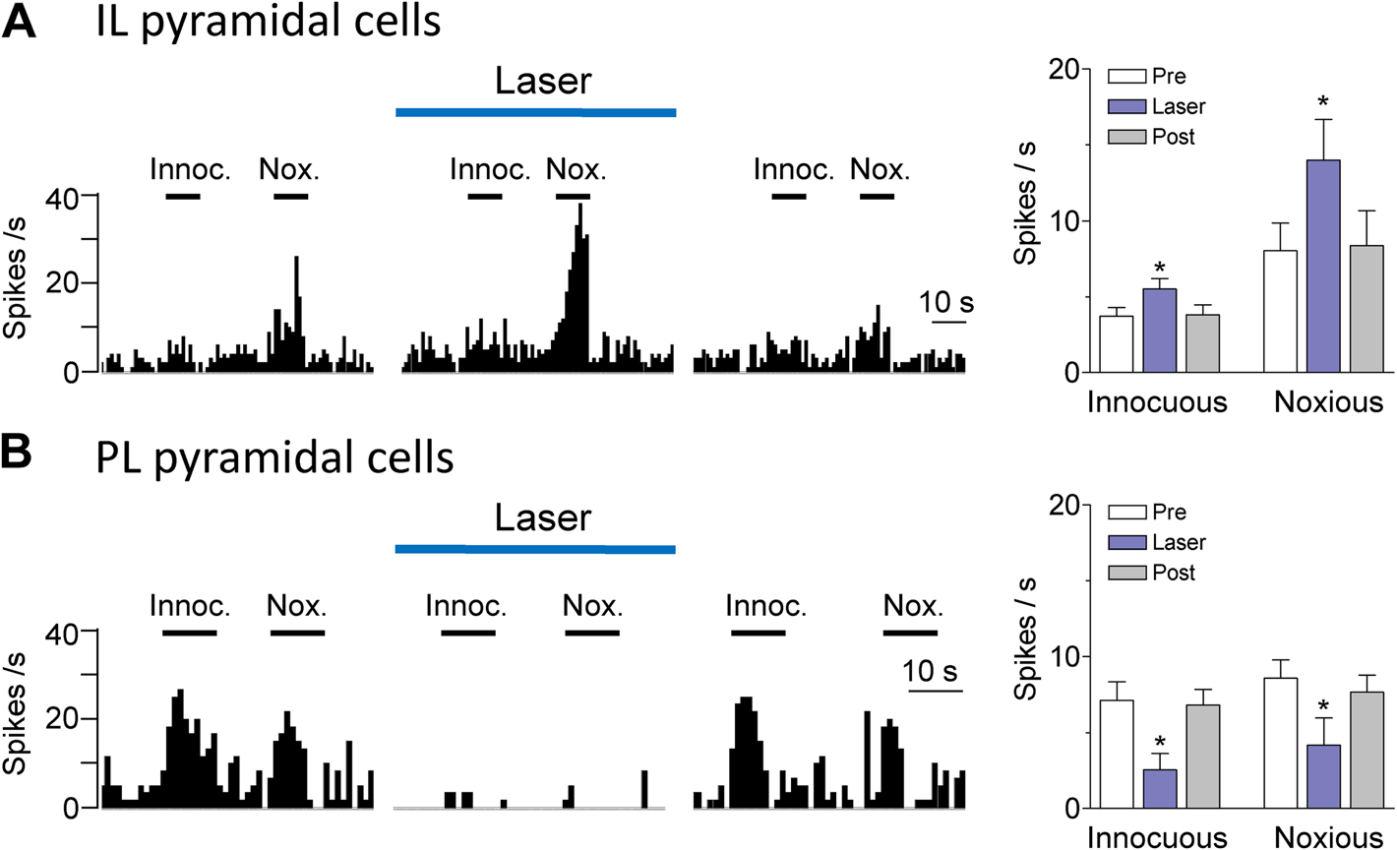
**Effects of optical activation on evoked activity.** Effect of optical stimulation on the responses of mPFC pyramidal cells to brief (10 s) innocuous (300 g/30 mm^2^) and noxious (2000 g/30 mm^2^) somatosensory stimuli (mechanical compression of the knee, see Methods). (**A**) Responses of IL pyramidal cells increased during optical stimulation (1 mW, 10 Hz) in IL. Left, Peristimulus time histograms (bin width, 1 s) show responses of an individual neuron to innocuous (Innoc.) and noxious (Nox.) stimuli (horizontal bars) before, during and after laser stimulation. Right, Bar histograms show summary data (means ± SE) for the sample of IL pyramidal cells tested (n = 8). Pre, before; Post, after laser stimulation. Background activity preceding each stimulus has been subtracted from the total activity during stimulation to obtain the net evoked response. (**B**) Responses of PL pyramidal cells decreased during optical stimulation (5 mW, 10 Hz) in IL. Same display as in (A). Bar histograms show summary data for the sample of PL pyramidal cells tested (n = 5). * P < 0.05 (compared to control before stimulation “Pre”, Bonferroni posttests).

The mPFC receives multisensory including nociceptive information, is particularly concerned with the affective value of a stimulus, and forms strong reciprocal connections with limbic forebrain structures such as the amygdala that provides value-based emotional information [32,33,52]. However the optogenetic modulation of sensory-evoked responses of mPFC neurons remains to be determined. Laser stimulation (1 mW, 10 Hz, 1–2 min) of Chr2 expressing IL neurons led to a significant (P < 0.05, Bonferroni posttests) increase of brief (10 s) evoked responses of IL pyramidal cells (n = 8 neurons tested; Figure 4A). The facilitatory effect was particularly pronounced for responses to high-intensity stimuli. The lower intensity of 1 mW was selected to avoid confounding strong increases in background activity (see Figure 3A). In contrast, optical stimulation (5 mW, 10 Hz, 1–2 min) of ChR2 expressing IL neurons decreased the responses of PL neurons to innocuous and noxious stimuli significantly (n = 5 neurons tested, P < 0.05, Bonferroni posttests; Figure 4B).

### Controls

**Figure 5:**
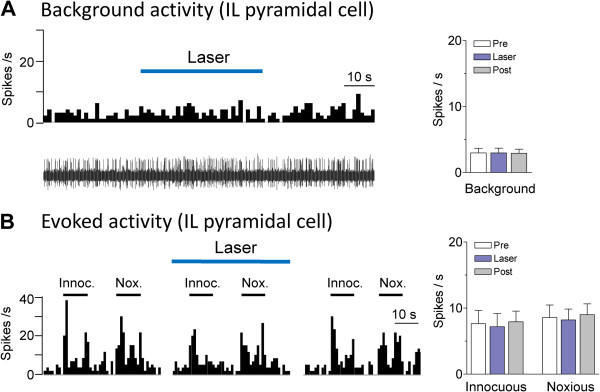
**Lack of effect of optical activation in animals treated with control virus.** Optical stimulation (5 mW, 10 Hz) in the IL of animals injected with a control virus that lacked the ChR2 sequence (rAAV5/CaMKIIa-EYFP) had no effect on background (**A**) and evoked (B) activity of IL pyramidal cells (n = 5 neurons). (**A**) Left, Peristimulus time histograms (PSTHs, bin width, 1 s) show number of action potentials (spikes/s) before, during and after laser stimulation in an individual neuron. Right, Bar histograms show summary data (means ± SE) for the sample of neurons tested (n = 5). (**B**) Left, PSTHs show responses of an individual neuron to innocuous (Innoc.) and noxious (Nox.) stimuli (horizontal bars) before, during and after laser stimulation. Right, Bar histograms show summary data (means ± SE) for the sample of neurons tested (n = 5). Background activity preceding each stimulus has been subtracted from the total activity during stimulation to obtain the net evoked response.

Optical stimulation (5 mW, 10 Hz, 2 min) in the IL of animals injected with a control virus that lacked the ChR2 sequence (rAAV5/CaMKIIa-EYFP) had no significant effect on IL pyramidal cells (n = 5 neurons; Figure 5).

## Discussion

The mPFC serves major executive functions and plays an important role in the modulation of emotional processing in subcortical centers such as the amygdala [53-58]. The present study advances our knowledge about function and manipulation of mPFC neurons in several ways.

First, optogenetic stimulation of ChR2-expressing excitatory neurons in the IL produced not only excitation of IL pyramidal cells but also increased their responsiveness to excitatory inputs driven by peripheral mechanical stimuli. mPFC neurons receive multisensory including somatosensory and nociceptive information [52]. Physiological nociceptive signals that normally activate mPFC output cells [32] likely serve protective functions such as attention, awareness and appraisal [59-61]. mPFC responses are believed to be related to the affective value of the stimulus which is consistent with the close reciprocal connections between mPFC and limbic forebrain structures such as the amygdala that provide emotion- and value-based information [32,33,51,52]. Our data suggest that optical stimulation provides a tool to increase pyramidal output not only through direct excitation but also by facilitating afferent input to drive pyramidal output.

Second, our results directly demonstrate an inverse interaction between infra- and prelimbic mPFC regions. Activation of IL output inhibited PL pyramidal cells, which likely involved feedforward inhibition [62]. Differential roles of infralimbic and prelimbic mPFC regions have been proposed with regard to their modulation of emotional processing associated with conditioned fear [15,23]. Specifically, IL plays a critical role in fear extinction likely through direct excitatory projections to a cluster of inhibitory neurons (intercalated cells) interposed between input and output regions of the amygdala [16]. Stimulation of IL facilitates extinction [63] and causes inhibition of amygdala output neurons [64]. Increased IL activity correlates with successful fear extinction [19,22] and decreased IL activity with extinction deficits [23-26]. In contrast, PL is involved in expression and renewal of fear [15,23]. Stimulation of the PL results in freezing behavior (Vidal-Gonzalez et al., 2006) and increased activation of amygdala input neurons [14]. Evoked responses of PL neurons correlate with fear conditioning and persistent activity with extinction deficits [65] whereas PL inactivation impairs fear expression [23]. Our data show that IL activation can inhibit PL output, suggesting that IL-mediated extinction mechanisms may not only involve direct interactions with the amygdala but also control of PL-driven facilitatory influences on fear expression.

The results of this study are significant because impaired mPFC function is associated with several neuropsychiatric disorders [1-8]. Modulating mPFC output may be utilized in the treatment of anxiety disorders such as PTSD [17,23,47] and optogenetic strategies to increase excitatory or inhibitory processes in the mPFC have been suggested as novel treatment strategies in neuropsychiatric disorders such as depression [66] and schizophrenia [67].

In a previous study we used electrical stimulation of labeled afferent fibers from the amygdala in the IL to show feedforward inhibition of PL pyramidal cells [32-34]. While these data are not fully comparable with the results of optical stimulation in the IL in the present study, they do agree on the presence of a circuit involving the IL that leads to inhibition of PL pyramidal cells. There are likely different cortical and extracortical sources that can engage feedforward inhibition of PL neurons (and IL neurons for that matter); these cannot easily be distinguished using electrical stimulation which may activate interneurons, pyramidal cells or fibers of passage. In contrast, optogenetics-based stimulation allows the activation of a defined population of neurons (IL pyramidal cells in the present study), which is one of the key advantages of this technology that we used here to show IL-induced inhibition of PL pyramidal cells.

As a technical consideration, the differential effects of optical stimulation on different neuronal populations and the lack of effect in animals injected with control virus argue against nonspecific confounding factors, including heating as the result of high light power, toxicity at high expression levels or long-term expression, and transient changes in ion balance [43]. The stimulus intensities used in this study (1, 5 and 10 mW) are well within the suitable range for optogenetic control despite the stimulation of a small volume of tissue [38]. We used the channelrhodopsin variant ChR2(H134R) that is widely used and can drive precise low-frequency spike trains [50].

## Conclusion

The electrophysiological in vivo results directly demonstrate the optogenetic modulation of mPFC activity in a region- and cell type-specific manner, which is significant because optogenetic tools may be useful in neuropsychiatric disorders associated with impaired mPFC function [43,68]. The selective, controlled, cell type-specific intervention can provide important insights into neurobiological mechanisms of complex brain functions and disorders.

Our results show an inverse relationship between IL and PL, suggesting that some IL functions may not only involve direct projections to subcortical limbic structures but also engage inhibitory control over PL output. The effects on background and evoked activity suggest that optical stimulation provides a tool to increase pyramidal output not only through direct excitation but also by facilitating the effectiveness of afferent drives.

## Abbreviations

ChR2: Channel rhodopsin 2; IL: Infralimbic; mPFC: Medial prefrontal cortex; PL: Prelimbic.

## Competing interests

The authors declare that they have no competing financial interests.

## Authors’ contributions

G.J. performed the experiments, analyzed data, provided figures and wrote the first draft of the manuscript. V.N. conceptualized the hypothesis, designed and supervised the experiments, directed the data analysis, and finalized the manuscript. All authors read and approved the manuscript.
